# Supplementary material for: An IL-21R STAT hypomorph circumvents functional redundancy in germinal centre responses
Source: eLife. 2026 Oct 23;15:RP109834. doi: 10.7554/eLife.109834 (PMC13694099; doi:10.7554/eLife.109834)
Supplement: Supplementary file 1 [file elife-109834-fig1-data1.pdf]

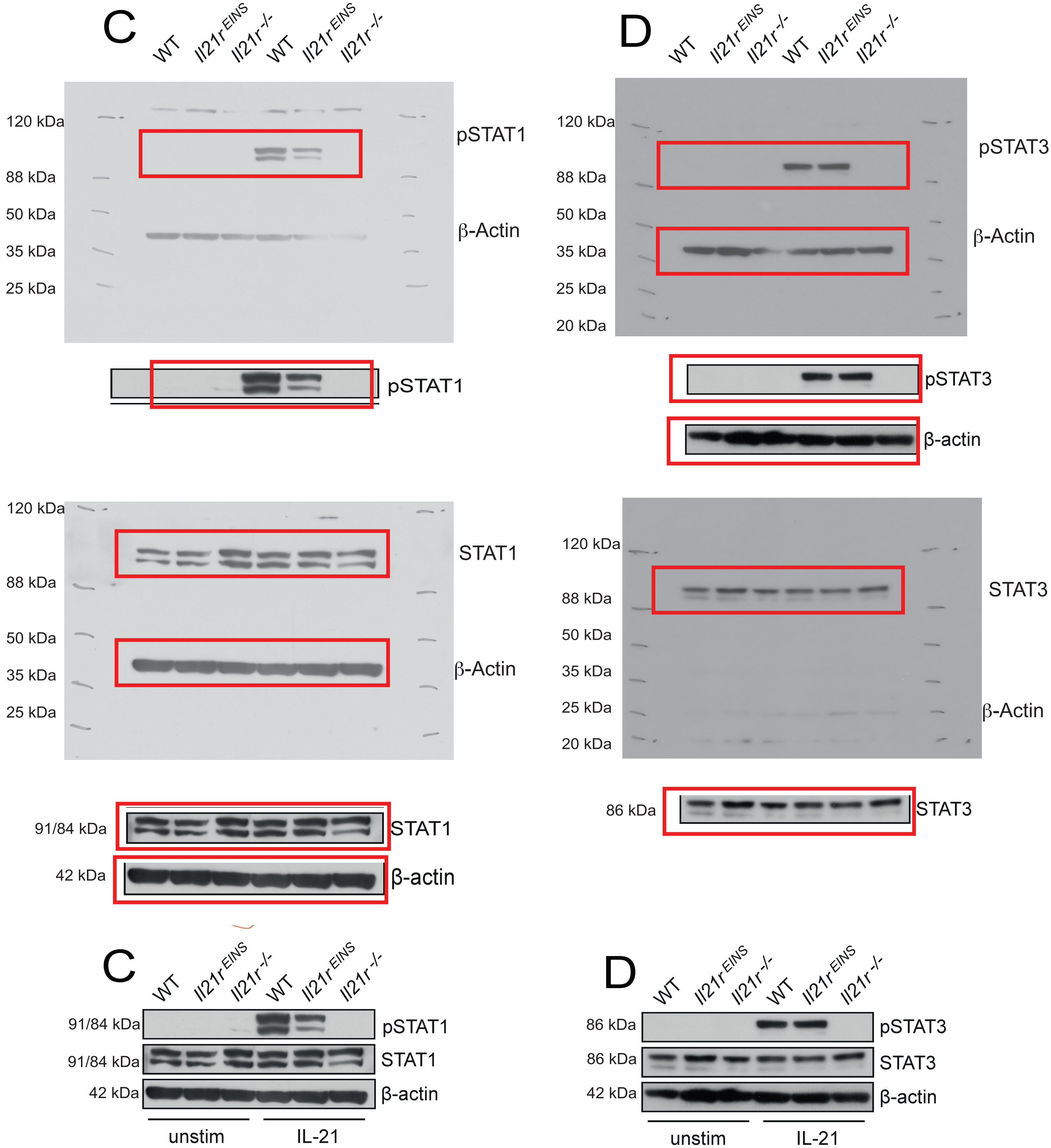
C
WT
Il21r EINS
Il21r-/-
WT
Il21r EINS
Il21r-/-
120 kDa
88 kDa
50 kDa
35 kDa
25 kDa
pSTAT1
β-Actin
pSTAT1
STAT1
β-Actin
91/84 kDa
STAT1
42 kDa
β-actin
D
120 kDa
88 kDa
50 kDa
35 kDa
25 kDa
20 kDa
pSTAT3
β-Actin
pSTAT3
β-actin
STAT3
β-Actin
86 kDa
STAT3
91/84 kDa
pSTAT1
STAT1
42 kDa
β-actin
unstim
IL-21
86 kDa
pSTAT3
STAT3
β-actin
unstim
IL-21
