# Supplementary material for: An IL-21R STAT hypomorph circumvents functional redundancy in germinal centre responses
Source: eLife. 2026 Oct 23;15:RP109834. doi: 10.7554/eLife.109834 (PMC13694099; doi:10.7554/eLife.109834)
Supplement: Supplementary file 2 [file elife-109834-mdarchecklist1.pdf]

eLife

Materials Design Analysis Reporting (MDAR) Checklist for Authors

The MDAR framework establishes a minimum set of requirements in transparent reporting mainly applicable to studies in the life sciences.

eLife asks authors to **provide detailed information within their article** to facilitate the interpretation and replication of their work. Authors can also upload supporting materials to comply with relevant reporting guidelines for health-related research (see EQUATOR Network), life science research (see the BioSharing Information Resource), or animal research (see the ARRIVE Guidelines and the STRANGE Framework; for details, see *eLife*'s Journal Policies). Where applicable, authors should refer to any relevant reporting standards materials in this form.

For all that apply, please note **where in the article** the information is provided. Please note that we also collect information about data availability and ethics in the submission form.

Materials:

Newly created materials	Indicate where provided: section/figure legend	N/A
The manuscript includes a dedicated "materials availability statement" providing transparent disclosure about availability of newly created materials including details on how materials can be accessed and describing any restrictions on access.	"Materials availability statement" provided in manuscript.	

Antibodies	Indicate where provided: section/figure legend	N/A
For commercial reagents, provide supplier name, catalogue number and RRID, if available.	Antibody suppliers, clone numbers and catalogue numbers are provided in the Methods sections “Immunofluorescence and FACS analyses,” “Immunohistochemistry” and “SDS Page and Immunoblotting.”<br>Flow Cytometry Antibodies and Reagents<br>B220/APC, clone RA3-6B2 - BD; catalog number: 561880.<br>B220/APC-Cy7, clone RA3-6B2 - BD; catalog number: 561102.<br>CD4/PE-Cy7, clone RM4-5 - BD; catalog number: 552775.<br>CD4/Alexa Fluor 488, clone RM4-5 - BD; catalog number: 557667.<br>CD21/FITC, clone 7G6 - BD; catalog number: 561769; legacy 553818.<br>CD23/PE, clone B3B4 - BD; catalog number: 553139.<br>CD25/APC, clone PC61 - BD; catalog number: 561048.	

	CD25/BV605, clone PC61 - BD; catalog number: 563061.<br>CD45.1/PE-Cy7, clone A20 - BD; catalog number: 560578.<br>CD45.2/APC-Cy7, clone 104 - BD; catalog number: 560694.<br>CXCR5/biotin, clone 2G8 - BD; catalog number: 551960.<br>GL7/PE, clone GL7 - BD; catalog number: 561530.<br>Fas/FITC, clone Jo2 - BD; catalog number: 561979.<br>IgG1/biotin, clone A85-1 - BD; catalog number: 550331.<br>Ly6G/Pacific Blue, clone 1A8 - BioLegend; catalog number: 127612<br>STAT1-pY701/Alexa Fluor 647, clone 4a - BD; catalog number: 612597.<br>STAT3-pY705/PE, clone 4/P-STAT3 - BD; catalog number: 612569.<br>STAT5-pY694/PerCP-Cy5.5, clone 47 - BD; catalog number: 560118.<br>IL-17A/APC-Cy7, clone TC11-18H10 - BD; catalog number: 560821.<br>CD8/APC, clone 53-6.7 - eBioscience; catalog number: 17-0081-82.<br>CD11b/FITC, clone M1/70 - eBioscience; catalog number: 11-0112-82.<br>CD11c/APC, clone N418 - eBioscience; catalog number: 17-0114-82.<br>CD19/eFluor450, clone 1D3 - eBioscience; catalog number: 48-0193-82.<br>CD38/PE-Cyanine5.5, clone 90 - eBioscience; catalog number: 15-0381-82.<br>CD44/APC-eFluor780, clone IM7 - eBioscience; catalog number: 47-0441-82.<br>ICOS/PE, clone C398.4A - eBioscience; catalog number: 12-9949-81.<br>NK1.1/PE, clone PK136 - eBioscience; catalog number: 12-5941-82.<br>PD-1/FITC, clone RMP1-30 - eBioscience; catalog number: 11-9981-82.<br>TCRβ/APC, clone H57-597 - eBioscience; catalog number: 17-5961-82.	

		TCRβ/FITC, clone H57-597 - eBioscience; catalog number: 11-5961-82.<br>TCRβ/PerCP-Cy5.5, clone H57-597 - eBioscience; catalog number: 45-5961-82.<br>Foxp3/eFluor450, clone FJK-16s - eBioscience; catalog number: 48-5773-82.<br>Immunohistochemistry<br>CD4/PE, clone RM4-5 - BD; catalog number: 553049.<br>IgG1/biotin, clone A85-1 - BD; catalog number: 550331.<br>IgD/Alexa Fluor 488, clone 11-26c.2a - BioLegend; catalog number: 405718.<br>Western Blotting<br>STAT1, clone 42H3 - Cell Signaling Technology; catalog number: 9175.<br>STAT3, clone F-2 - Santa Cruz; catalog number: sc-8019.<br>β-actin, clone AC-74 - Sigma-Aldrich; catalog number: A2228.<br>Phospho-STAT1, clone D4A7 - Cell Signaling Technology; catalog number: 7649.<br>Phospho-STAT3, clone 3E2 - Cell Signaling Technology; catalog number: 9138.<br>Goat anti-rabbit IgG-HRP - Santa Cruz; catalog number: SC-2004.<br>Goat anti-mouse IgG-HRP - Thermo Fisher; catalog number: 31439.<br>Rabbit anti-goat IgG-HRP - Thermo Fisher; catalog number: 31433.<br>Additional Antibodies<br>Neutralising anti-IL-6, clone MP5-20F3 - Bio X Cell; catalog number: BE0046.<br>Anti-CD3, clone 145-2C11 - BD; catalog number: 553057.<br>Anti-CD28, clone 37.51 - eBioscience; catalog number: 16-0281-85<br>Anti-IL-4 - clone 11B11 - BD; catalog number: 554387.<br>Anti-IFNγ - clone XMG1.2 - BD; catalog number: 554408<br>IL-21R-Fc - Manufactured in-house as demonstrated previously.	

DNA and RNA sequences	Indicate where provided: section/figure legend	N/A
Short novel DNA or RNA including primers, probes: Sequences should be included or deposited in a public repository.		N/A

Cell materials	Indicate where provided: section/figure legend	N/A
Cell lines: Provide species information, strain. Provide accession number in repository OR supplier name, catalog number, clone number, OR RRID.		N/A
Primary cultures: Provide species, strain, sex of origin, genetic modification status.	Primary splenocytes were obtained from the mouse strains described in the “Mice” section and used for flow cytometry, stimulation and immunoblotting.	

Experimental animals	Indicate where provided: section/figure legend	N/A
Laboratory animals or Model organisms: Provide species, strain, sex, age, genetic modification status. Provide accession number in repository OR supplier name, catalog number, clone number, OR RRID.	The study used *Mus musculus*. The mouse strains and models included C57BL/6 (B6) wild-type mice, Ly5.1 congenic mice on a B6 background, RAG1−/− mice bred to B6 N10, Il21r−/− mice obtained at B6 N6 and backcrossed to N12, and Il21rEINS mice on a C57BL/6 background. The Il21rEINS model carries an ENU-induced Asp375Val point mutation in *Il21r*. C57BL/6, Ly5.1 congenic and RAG1−/− mice were purchased from the Animal Research Centre, Perth, Australia. Il21r−/− mice were obtained from Dr Warren Leonard via Dr Mark Smyth, and Il21rEINS mice were obtained from the Australian Phenomics Facility, ANU. Mice were age-matched littermates aged 8–14 weeks, both sexes were used and mice were housed under specific pathogen-free conditions. Location: Materials and Methods, “Mice”; the *Il21rEINS* mutation is additionally described in the	

	Results and Figure 1 legend.	
Animal observed in or captured from the field: Provide species, sex, and age where possible.		N/A

**Plants and microbes**	**Indicate where provided: section/figure legend**	**N/A**
Plants: provide species and strain, ecotype and cultivar where relevant, unique accession number if available, and source (including location for collected wild specimens).		N/A
Microbes: provide species and strain, unique accession number if available, and source.		N/A

**Human research participants**	**Indicate where provided: section/figure legend) or state if these demographics were not collected**	**N/A**
If collected and within the bounds of privacy constraints report on age, sex, gender and ethnicity for all study participants.		**N/A**

Design:

**Study protocol**	**Indicate where provided: section/figure legend**	**N/A**
If the study protocol has been pre-registered, provide DOI. For clinical trials, provide the trial registration number OR cite DOI.		N/A

**Laboratory protocol**	**Indicate where provided: section/figure legend**	**N/A**
Provide DOI OR other citation details if detailed step-by-step protocols are available.		N/A

**Experimental study design (statistics details) ***		
**For in vivo studies: State whether and how the following have been done**	**Indicate where provided: section/figure legend. If it could have been done, but was not,**	**N/A**

	**write “not done”**	
Sample size determination	The numbers of mice per group and the numbers of independent experimental repeats are reported in the relevant figure legends. For example, sample sizes range from *n* = 4–8 per group, with two or three experimental repeats. Figure legends for Figures 1–7; Methods section, “Data analysis and Statistics.” Resource equation approach: Based on this approach, the acceptable range of degrees of freedom (DF) for the error term in an analysis of variance (ANOVA) is between 10 to 20. n = DF/k + 1 where N = total number of subjects, k = number of groups, and n = number of subjects per group. Sample size; 20/4+1=5 animals/group per experiment.	
Randomisation	Randomisation not reported.	
Blinding	Blinding during animal allocation, sample processing, flow-cytometric analysis and data analysis was not reported in the manuscript.	
Inclusion/exclusion criteria	Inclusion criteria were age-matched and sex-matched (male and female) littermate mice aged 8–14 weeks housed under specific pathogen-free conditions. Predefined exclusion criteria were not reported. Location: Materials and Methods, “Mice,” and the relevant figure legends.	

**Sample definition and in-laboratory replication**	**Indicate where provided: section/figure legend**	**N/A**
State number of times the experiment was replicated in the laboratory.	The manuscript reports two to four experimental repeats depending on the figure. Individual mice are biological replicate. Location: Relevant figure legends.	
Define whether data describe technical or biological replicates.	Each individual mouse represents a biological replicate. The number	

	of experimental repeats is reported in the relevant figure legends.	

**Ethics**	**Indicate where provided: section/submission form**	**N/A**
Studies involving human participants: State details of authority granting ethics approval (IRB or equivalent committee(s), provide reference number for approval.		N/A
Studies involving experimental animals: State details of authority granting ethics approval (IRB or equivalent committee(s), provide reference number for approval.	The Garvan Institute and St. Vincent's Hospital Animal Ethics Committee granted permission to perform animal experiments (15_20). Animals were housed under specific pathogen-free conditions and handled in accordance with the Australian code of practice for the care and use of animals for scientific purposes.	
Studies involving specimen and field samples: State if relevant permits obtained, provide details of authority approving study; if none were required, explain why.		N/A

**Dual Use Research of Concern (DURC)**	**Indicate where provided: section/submission form**	**N/A**
If study is subject to dual use research of concern regulations, state the authority granting approval and reference number for the regulatory approval.		N/A

Analysis:

**Attrition**	**Indicate where provided: section/figure legend**	**N/A**
Describe whether exclusion criteria were pre-established. Report if sample or data points were omitted from analysis. If yes, report if this was due to attrition or intentional exclusion and provide justification.	No exclusions, attrition or omitted data points were used.	

**Statistics**	**Indicate where provided: section/figure legend**	**N/A**
Describe statistical tests used and justify choice of tests.	Statistical analyses were performed using GraphPad Prism.	

	Data are presented as individual mice with mean ± SD. Comparisons between two groups were performed using an unpaired Student's *t*-test or paired t-test for mixed bone marrow chimera analyess. Comparisons involving more than two groups were performed using one-way ANOVA followed by Bonferroni's multiple-comparisons test. Two-way ANOVA with multiple-comparisons testing was used where indicated in the figure legends.<br><br>One-way ANOVA with Bonferroni's multiple-comparisons test was used for Figures 1, 2, 3, 6A–F and 7. Student's *t*-tests were used for Figures 4 and 5, and two-way ANOVA was used for Figure 6G–H. Statistical significance was indicated as $p < 0.05$, with $p < 0.05$, $p < 0.01$, $p < 0.001$ and $p < 0.0001$ represented by one, two, three and four asterisks, respectively. Location: Methods section, "Data analysis and Statistics," and the relevant figure legends.	

**Data availability**	**Indicate where provided: section/submission form**	**N/A**
For newly created and reused datasets, the manuscript includes a data availability statement that provides details for access (or notes restrictions on access).		N/A
When newly created datasets are publicly available, provide accession number in repository OR DOI and licensing details where available.		N/A
If reused data is publicly available provide accession number in repository OR DOI, OR URL, OR citation.		N/A

**Code availability**	**Indicate where provided: section/figure legend**	**N/A**

For any computer code/software/mathematical algorithms essential for replicating the main findings of the study, whether newly generated or re-used, the manuscript includes a data availability statement that provides details for access or notes restrictions.		N/A
Where newly generated code is publicly available, provide accession number in repository, OR DOI OR URL and licensing details where available. State any restrictions on code availability or accessibility.		N/A
If reused code is publicly available provide accession number in repository OR DOI OR URL, OR citation.		N/A

Reporting:

The MDAR framework recommends adoption of discipline-specific guidelines, established and endorsed through community initiatives.

**Adherence to community standards**	**Indicate where provided: section/figure legend**	**N/A**
State if relevant guidelines (e.g., ICMJE, MIBBI, ARRIVE, STRANGE) have been followed, and whether a checklist (e.g., CONSORT, PRISMA, ARRIVE) is provided with the manuscript.	The manuscript contains the relevant information in the Methods and figure legends, but randomisation and blinding were not reported, and exclusion criteria were not stated.	

---

* We provide the following guidance regarding transparent reporting and statistics; we also refer authors to Ten common statistical mistakes to watch out for when writing or reviewing a manuscript.

Sample-size estimation

- You should state whether an appropriate sample size was computed when the study was being designed
- You should state the statistical method of sample size computation and any required assumptions
- If no explicit power analysis was used, you should describe how you decided what sample (replicate) size (number) to use

Replicates

- You should report how often each experiment was performed
- You should include a definition of biological versus technical replication
- The data obtained should be provided and sufficient information should be provided to indicate the number of independent biological and/or technical replicates
- If you encountered any outliers, you should describe how these were handled
- Criteria for exclusion/inclusion of data should be clearly stated
- High-throughput sequence data should be uploaded before submission, with a private link for reviewers provided (these are available from both GEO and ArrayExpress)

Statistical reporting

- Statistical analysis methods should be described and justified
- Raw data should be presented in figures whenever informative to do so (typically when N per group is less than 10)
- For each experiment, you should identify the statistical tests used, exact values of N, definitions of center, methods of multiple test correction, and dispersion and precision measures (e.g., mean, median, SD, SEM, confidence intervals; and, for the major substantive results, a measure of effect size (e.g., Pearson's r, Cohen's d)
- Report exact p-values wherever possible alongside the summary statistics and 95% confidence intervals. These should be reported for all key questions and not only when the p-value is less than 0.05.

Group allocation
- Indicate how samples were allocated into experimental groups (in the case of clinical studies, please specify allocation to treatment method); if randomization was used, please also state if restricted randomization was applied
- Indicate if masking was used during group allocation, data collection and/or data analysis
