# Supplementary material for: An IL-21R STAT hypomorph circumvents functional redundancy in germinal centre responses
Source: eLife. 2026 Oct 23;15:RP109834. doi: 10.7554/eLife.109834 (PMC13694099; doi:10.7554/eLife.109834)
Supplement: Peer review history

# Editor report: eLife Assessment

**DOI:** 10.7554/eLife.109834.3.sa0

**Authors:** Tomohiro Kurosaki

**Affiliations:** The University of Osaka Japan

**Keywords:** Incomplete, Valuable

These results add to a growing body of literature demonstrating a role for IL-21 in T follicular helper cell expansion and differentiation, as well as in germinal center formation and maintenance. The authors use an IL21R ENU-induced mutant, together with complementary in vitro and in vivo experiments, to investigate the roles of STAT1 and STAT3 signaling downstream of IL-21R. The experimental approach is **valuable**, but the main conclusion is **incompletely** supported by the evidence, as the mutant exhibits a broad reduction in STAT signaling rather than a selective impairment of STAT1 signaling.

# Referee report: Reviewer #1 (Public review)

**DOI:** 10.7554/eLife.109834.3.sa1

Summary:

King and colleagues generated a mouse with a point mutation in IL21R and investigate the influence on IL-21-mediated T and B cell activation and differentiation. They find that mutant mice show a reduced T and B cell response with CD4 T cell differentiation into T follicular helper cells being primarily affected.

Strengths:

The authors combine in vitro and in vivo analysis, including bone-marrow chimeric mice.

Weaknesses:

The effect of the IL21R EINS mutant does not specifically affect STAT1, as clearly shown in Figure 1 H, I. Particularly at lower doses of IL21 which may be more relevant in vivo, the effects are very similar. A second key weakness is the very small Tfh response, a not very clear PD-1 and CXCR5 staining to identify Tfh and a lack of a steady-state (prior to immunisation) comparison of Tfh numbers in the different mouse strains. The latter makes it impossible to know what fraction of the response is antigen-specific.

Comments on revised version.

Thank you for responding to some of my suggestions/comments.

Unfortunately, these responses do not address the concerns raised by the other reviewer and me (see 'weaknesses'). The argument that statistical analysis shows that there is no difference in p-STAT3/5 does not make any sense - it simply reflects the absence of sufficient statistical power.

No further experiments have been performed, and the authors didn't appropriately adjust the conclusions to reflect the limitations of the study.

To provide an example, the authors decided to keep the title 'An IL-21R hypomorph circumvents functional redundancy to define STAT1 signalling in germinal center responses' although both reviewers clearly highlighted that no conclusion about STAT1 can be made and the authors responded that 'We agree that further experiments are needed to definitively show that the effect is attributable to reduced STAT1 activation alone. Rescue experiments will be a focus of future experiments.'

These rescue experiments (and additional analysis of the Tfh response) are essential to allow firms conclusions to be made.

In its current form, the manuscript is unfortunately misleading.

# Referee report: Reviewer #2 (Public review)

**DOI:** 10.7554/eLife.109834.3.sa2

Summary:

In the manuscript, "An IL-21R hypomorph circumvents functional redundancy to define STAT1 signaling in germinal center responses," Cecile King and colleagues identify a cytoplasmic site of the IL-21 receptor that differentially regulates STAT1 and STAT3 activation upon IL-21 stimulation. They further examine the immunological consequences of this site-specific alteration on Tfh differentiation and Tfh-dependent humoral immunity, raising important questions about how gene-knockout models may obscure nuanced functional roles of signaling molecules.

Strengths:

The study convincingly highlights a non-redundant role for STAT1 downstream of IL-21-IL-21R signaling in the Tfh differentiation pathway. This conclusion is supported by in vitro analyses of STAT1 and STAT3 activation in CD4 T cells stimulated with IL-21 or IL-6; by in vivo assessments of Tfh and germinal center B cell responses in WT and IL21R-EINS mutant mice, including bone-marrow chimera systems; and by investigating the expression of Tfh-related molecules in WT versus IL21R-EINS CD4 T cells.

Weaknesses:

Although the experiments were carefully executed with appropriate controls, a key question remains unresolved: whether the Tfh differentiation defect in IL21R-EINS mice is directly attributable to reduced STAT1 activation. Rescue experiments that restore STAT1 signaling in IL21R-EINS TCR-transgenic CD4 T cells would provide strong evidence linking the mutation to impaired STAT1 activation and, consequently, defective Tfh differentiation. Without such evidence, it remains formally possible that additional, uncharacterized mutations introduced during ENU mutagenesis contribute to the phenotypes observed, particularly given the discrepancies between IL21R knockout and IL21R-EINS mutant mice.

Comments on revised version.

The revised manuscript failed to address the key question, whether the Tfh differentiation defect in IL21R-EINS mice results from the reduced STAT1 activation in CD4 T cells.

# Author comment: Author response

**DOI:** 10.7554/eLife.109834.3.sa3

**Authors:** Christoph Jandl, Joanna Warren, Samantha Owens, Marcel Batten, Howard Wang, Cecile King

**Affiliations:** Garvan Institute of Medical Research Darlinghurst Australia; Garvan Institute of Medical Research Darlinghurst Australia; UNSW Sydney Kensington Australia; Garvan Institute of Medical Research Darlinghurst Australia; UNSW Sydney Kensington Australia; UNSW Sydney Kensington Australia

The following is the authors’ response to the original reviews.

**Public Reviews:**

**Reviewer #1 (Public review):**

Summary:

King and colleagues generated a mouse with a point mutation in IL21R and investigated the influence on IL-21-mediated T and B cell activation and differentiation. They found that mutant mice show a reduced T and B cell response, with CD4 T cell differentiation into T follicular helper cells being primarily affected.

Strengths:

The authors combined in vitro and in vivo analysis, including bone-marrow chimeric mice.

Weaknesses:

The effect of the IL21R EINS mutant does not specifically affect STAT1, as clearly shown in Figure 1 H, I. Particularly at lower doses of IL21, which may be more relevant in vivo, the effects are very similar. A second key weakness is the very small Tfh response, a not very clear PD-1 and CXCR5 staining to identify Tfh, and a lack of a steady-state (prior to immunisation) comparison of Tfh numbers in the different mouse strains. The latter makes it impossible to know what fraction of the response is antigen-specific.

**Reviewer #2 (Public review):**

Summary:

In the manuscript, "An IL-21R hypomorph circumvents functional redundancy to define STAT1 signaling in germinal center responses," Cecile King and colleagues identify a cytoplasmic site of the IL-21 receptor that differentially regulates STAT1 and STAT3 activation upon IL-21 stimulation. They further examine the immunological consequences of this site-specific alteration on Tfh differentiation and Tfh-dependent humoral immunity, raising important questions about how geneknockout models may obscure nuanced functional roles of signaling molecules.

Strengths:

The study convincingly highlights a non-redundant role for STAT1 downstream of IL-21-IL-21R signaling in the Tfh differentiation pathway. This conclusion is supported by in vitro analyses of STAT1 and STAT3 activation in CD4 T cells stimulated with IL-21 or IL-6; by in vivo assessments of Tfh and germinal center B cell responses in WT and IL21R-EINS mutant mice, including bonemarrow chimera systems; and by investigating the expression of Tfh-related molecules in WT versus IL21R-EINS CD4 T cells.

Weaknesses:

Although the experiments were carefully executed with appropriate controls, a key question remains unresolved: whether the Tfh differentiation defect in IL21R-EINS mice is directly attributable to reduced STAT1 activation. Rescue experiments that restore STAT1 signaling in IL21R-EINS TCR-transgenic CD4 T cells would provide strong evidence linking the mutation to impaired STAT1 activation and, consequently, defective Tfh differentiation. Without such evidence, it remains formally possible that additional, uncharacterized mutations introduced during ENU mutagenesis contribute to the phenotypes observed, particularly given the discrepancies between IL21R knockout and IL21R-EINS mutant mice.

We agree that further experiments are needed to definitively show that the effect is attributable to reduced STAT1 activation alone. Rescue experiments will be a focus of future experiments.

**Recommendations for the authors:**

**Reviewer #1 (Recommendations for the authors):**

(1) Figure1

I would recommend changing the conclusion in Line 141 to 'potentially less affected' rather than unaffected as there is a clear and very consistent effect on pSTAT3 in every IL21 dose tested. Also, it seems that much more IL21 is required to induce STAT1 phosphorylation, which may explain the increased effect of the EINS mutant on this signalling pathway. Similarly, for the STAT5 data in Figure S1, there is very little phosphorylation beyond baseline phosphorylation (unstimulated), but there is a clear and consistent reduction in pSTAT5 in the EINS mutants.

Neither pSTAT3 nor pSTAT5 were significantly different between WT and IL21rEINS cells in either the percentage or MFI. We have edited line 141 to state “the levels of phosphorylated STAT3 in CD4+ T cells were significantly less affected by the Il21r^EINS^ mutation (Fig. 1I).”

A minor point: Why is the MFI in IL21R-/- mice at 300 in panel C and at 200 in panel E? How representative is the reduced baseline pSTAT5 in IL21r-/- mice?

This is likely due to machine voltage during acquisition in a different experiment.

Collectively, I would suggest concluding from that data that the EINS mutation affects IL21R signaling, which results in reduced STAT1, 3, and 5 phosphorylation, with pSTAT1 being most strongly reduced, particularly at high IL21 concentration.

Please also see response to above comment. Since neither pSTAT3 nor pSTAT5 were significantly different between WT and IL21rEINS cells, the data does not support that conclusion.

All subsequent data therefore do not investigate the effect of the EINS mutation on STAT1, but on overall reduced IL21R signalling. This needs to be considered when interpreting the data. For example, the text in lines 171, 172, and 174 should be adjusted as the effect is neither only dependent on STAT1 nor is STAT3 signalling intact.

Please also see responses above. It is possible that a different method for detection of phosphorylated STAT3 and STAT5 could have looked more closely into the effect at very low concentrations of IL-21. However, our findings using Westen Blot and flow cytometry only observed a significant difference in STAT1 activation. We have edited our sentence on line 333 to state “response in the presence of an IL-21 receptor mutant that predominantly affects IL-21 activation of STAT1”.

A key signalling pathway downstream of IL21R is AKT and S6 phosphorylation. It would be important to also investigate the effect of the EINS mutation on these pathways.

We agree and this will be a focus of future experiments.

(2) Figure 2

PNA or BCL-6 staining would be preferable to identify GC in Figure 1A, but the flow cytometry data in Figure 3 are convincing, so this is not absolutely necessary.

Also, no conclusions can be made here about STAT1 specifically, and there could be other reasons why Tfh are slightly and temporarily reduced in EINS mice.

(3) Figure 3

Line 200: Please explain what is meant by 'despite an expansion of the IgG1 FAs B cell population on day5, the percentages of EINS IgG1* GC B cells were significantly lower (Fig. 3F). I cannot see any expansion of IgG1 FAS B cells, nor can I see a specific effect on day 7. Both total GC B cells and IgG1 GC B cells are similarly affected throughout the response. Some data points may not reach statistical significance, but the trend is very clear.

Figure 3E shows the percentage of GC B cells increasing from day 3 to day 7 in WT and from day 3 to day 5 in IL21rEINS, with significant differences between WT and IL21rEINS on days 5 and 7. In Figure 3F he percentage of IgG1+ GC B cells increase from day 3 to day 14 in both genotypes, with a significant difference between IL21rEINS on day 7. We have edited to manuscript to state “Despite an increase in the IgG1^+^ FAS^+^ B cell population from day 3 in response to immunization, the percentages of Il21r^EINS^ IgG1+ GC B cells were significantly lower relative to WT cells 7 days after SRBC immunisation (Fig. 3F).”

(4) Figure 4

How many days after SRBC immunization was the analysis done?

The data show an intrinsic role of IL21R signalling to Tfh development, which may include a role for STAT1. The absence of any effect on GC B cells is somewhat surprising. Chimeric and irradiated mice sometimes mount poor immune responses, and GC B cell numbers are very low. What is the frequency of GC B cells in non-immunized mice? This would be important to know if the mice responded at all, and if they did, if the 'baseline' of GC activity differs.

As stated in the figure legend for Figure 4 – on day 7 “Mixed BM chimaeras were reconstituted with equal ratios of WT CD45.1+ BM cells and Il21rEINS CD45.2+ BM cells. 8 weeks after transfer, the mice were immunized with SRBC and analysed 7days later.

(5) Figure 7

Please highlight that while only IL21R-/- mice showed a significant difference in the frequency of Tfh, a similar trend was observed in WT and IL21Reins mice. The data spread is smaller in the IL21R-deficient mice, facilitating statistical significance. As throughout the manuscript, this is not a STAT1 IL-21R mutant; it is a mutant with reduced IL21R signalling. In fact, the finding that IL-6 does not compensate for the EINS mutation may suggest that STAT1 plays a minor role in the biological effects observed.

We can only report on the statistical significance of the data we have.

(6) Other comments

Figure 1 F/G. I think the Y axis should read pSTAT1 and pSTAT3, respectively.

Thank you, we have corrected the graph accordingly.

Figure S1A. Please change the order of WT, IL21EINS, and IL21R-/- to match the main figures (IL21R-/- last). Currently, A, B, and D have a different order, but C is like the main figures.

The figure panels are aligned to show media, then either IL-2 or IL-6 and then IL21.

Please provide complete flow cytometry gating strategies for all figures.

Flow cytometry dating for P-STAT1 and p-STAT3 is shown in figure 1, for Tfh cells and Tfr cells in Figure 2, 4 and 5. Please also see supplementary figures for T cell gating and methods for detailed description of antibodies and dilutions used for immunostaining.

**Reviewer #2 (Recommendations for the authors):**

Line 332 requires revision.

We have edited the final sentence to state” Taken together these findings demonstrate that, despite the strong ability of IL-6 to activate STAT1, IL-6 is ineffective at fully compensating for the germinal centre response in the presence of an IL-21 receptor mutant that predominantly affects IL-21 activation of STAT1.”
